# Mutant p53-Mediated Tumor Secretome: Bridging Tumor Cells and Stromal Cells

**DOI:** 10.3390/genes15121615

**Published:** 2024-12-17

**Authors:** Lei Qiu, Zelong Ma, Xiaoming Wu

**Affiliations:** Laboratory of Molecular Genetics of Aging & Tumor, Medical School, Kunming University of Science and Technology, Chenggong Campus, 727 South Jingming Road, Kunming 650500, China; avalon1011@163.com (L.Q.); mazeloong@163.com (Z.M.)

**Keywords:** mutant p53, tumor secretome, tumor microenvironment, secretory pathway, tumor immunization

## Abstract

The tumor secretome comprises the totality of protein factors secreted by various cell components within the tumor microenvironment, serving as the primary medium for signal transduction between tumor cells and between tumor cells and stromal cells. The deletion or mutation of the *p53* gene leads to alterations in cellular secretion characteristics, contributing to the construction of the tumor microenvironment in a cell non-autonomous manner. This review discusses the critical roles of mutant p53 in regulating the tumor secretome to remodel the tumor microenvironment, drive tumor progression, and influence the plasticity of cancer-associated fibroblasts (CAFs) as well as the dynamics of tumor immunity by focusing on both secreted protein expression and secretion pathways. The aim is to provide new insights for targeted cancer therapies.

## 1. Introduction

Cancer, one of the most formidable diseases faced by humanity in recent decades, claims numerous lives each year and poses a severe threat to human health. The majority of cancers are solid tumors originating from epithelial tissues, including those in the lungs, breasts, prostate, colon, pancreas, and ovaries. Despite significant advancements in diagnostic and therapeutic tools in recent years, the ultimate effectiveness of cancer treatment remains limited [1]. Tumors are dynamic systems in constant evolution, characterized by genetic and epigenetic changes within tumor cells and the dynamic interactions between tumors and their surrounding microenvironment. Reports indicate that the crosstalk between tumor cells and stromal cells during treatment forms a complex tumor microenvironment, playing a critical role in tumor recurrence and resistance mechanisms [2,3]. The tumor suppressor *p53*, known as the “guardian of the genome,” was initially classified as an oncogene but is now recognized as one of the most crucial protective factors in the human genome. The p53 protein, encoded by this gene, plays a key role in maintaining genomic stability [4,5,6,7]. Under normal circumstances, *p53* is activated in response to various types of cellular damage, including DNA damage, oxidative stress, and oncogenic signals. As a transcription factor, *p53* initiates DNA repair processes and regulates cell cycle arrest, apoptosis, and senescence signaling pathways to maintain genomic stability and prevent malignant transformation of cells [7,8,9,10,11]. For example, *p53* can transcriptionally activate *p21*, inhibiting cyclin-dependent kinases, leading to G1 phase cell cycle arrest. Additionally, *p53* can induce apoptosis by activating pro-apoptotic genes such as BAX and PUMA [12].

*Tp53* plays a crucial role in suppressing tumor growth. To overcome *p53*-mediated tumor suppression, cancer cells have evolved various strategies to eliminate *p53* function, promoting their growth and development. The most direct approach is through *Tp53* gene mutations. Cancer genome sequencing projects have identified *p53* mutations in nearly 50% of human tumors [13,14,15,16]. Known *p53* mutations include missense mutations, as well as deletions, truncations, and frameshift mutations [14,16]. Approximately 80% of *p53* missense mutations occur in specific bases within the DNA-binding domain of the *p53* gene, with R175, G245, R248, R249, R273, and R282 being known as *p53* hotspot mutations [16]. These missense mutants lose the ability to bind to established *p53* response DNA elements and initiate their intrinsic tumor-suppressive functions (loss of function—LOF). Furthermore, some missense mutants bind to wild-type p53 proteins expressed by non-mutated alleles, inactivating them (dominant-negative effect, DN) and acquiring new tumor-promoting functions (gain of function, GOF), thereby promoting tumor growth, survival, development, and metastasis through various mechanisms [17,18,19,20,21]. Concurrently, *p53* mutations can alter the types and secretion levels of proteins within the tumor [22], regulate the biochemical and biomechanical properties of the extracellular matrix, and modulate communication between tumor cells and between tumor cells and stromal cells [23]. The tumor microenvironment (TME) is a complex ecosystem composed of various non-malignant cell types, including vascular endothelial cells, immune cells (such as macrophages, T cells, natural killer cells), fibroblasts, and abundant extracellular matrices (ECMs). These components interact with tumor cells through the secretion of a series of bioactive molecules, such as growth factors, cytokines/chemokines, proteases, and angiogenic factors, constructing a complex microenvironment that supports tumor growth, invasion, and metastasis [24,25]. Several biological pathways, including Smad, PI3K, Jak/Stat, NF-κB, MAPK, CXCR2, and IL-1, have been shown to coordinate this intricate crosstalk system [26,27,28,29,30,31,32,33].

The tumor secretome comprises various proteins secreted by tumor cells and other components within the microenvironment [34,35,36]. These proteins affect tumor biology through mechanisms such as regulating intercellular communication, promoting angiogenesis, altering immune response patterns, and participating in metabolic reprogramming to adapt to changing environmental conditions [24,25]. The types and secretion levels of proteins released by tumor cells vary based on the tumor type and development stage [37,38,39,40,41,42,43]. Factors such as genetic mutations (e.g., *p53* mutations), hypoxia, nutritional status, inflammatory response, and therapeutic interventions influence the composition of the tumor secretome, thereby altering overall tumor progression.

This review will discuss the roles of mutated *p53* in regulating the tumor secretome and remodeling the tumor microenvironment, driving tumor progression through both protein expression and secretion pathways. This is a key mechanism by which mutant *p53* regulates tumor progression through cell non-autonomous pathways.

## 2. Mutant p53 Regulates the Expression of Secreted Proteins

**Figure 1:**
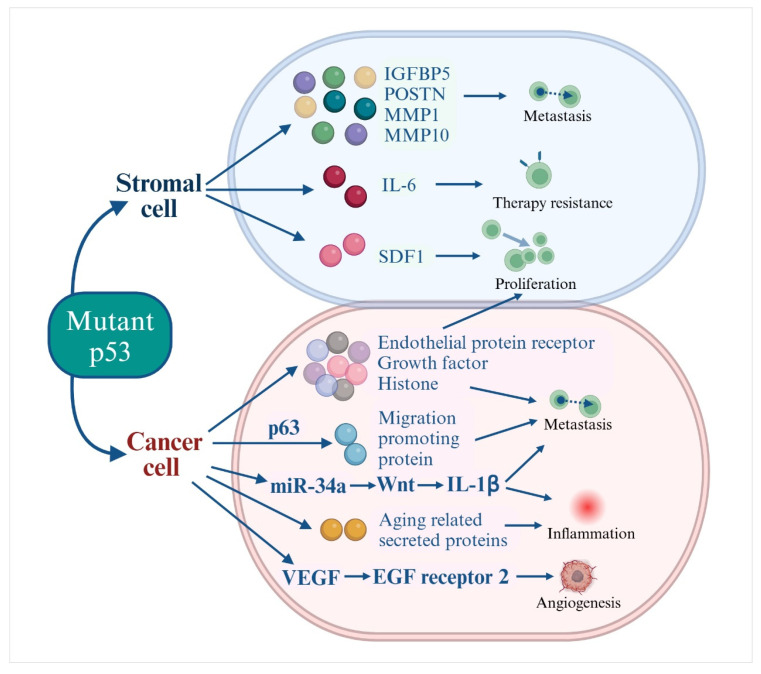
Mutant p53 regulates the secretome of stromal and tumor cells to promote tumor progression.

Numerous research findings indicate that *TP53* mutations not only alter the tumor-suppressive secretome driven by wild-type *p53* into a tumor-promoting profile but also directly participate in the regulation of secreted protein expression following mutation. In various tumor cell components, mutant *p53*-regulated secreted proteins perform distinct functions (Figure 1).

### 2.1. Mutant p53 Regulates the Expression of the Secretome in Tumor Cells

In tumor cells, the stability of mutant p53 proteins is elevated, which can upregulate the transcription of secreted proteins through three mechanisms: (1) mutant p53 directly enhances the transcription of secreted proteins; (2) mutant p53 indirectly promotes the transcription of secreted proteins by binding to other transcription factors; (3) mutant p53 regulates the transcription of secreted proteins indirectly through micro-RNAs, accelerating tumor progression [44].

Most studies indicate that mutant p53 is often recruited by other transcription factors to their respective target genes, thereby gaining specific functional activation [17,20,45,46,47]. However, an earlier report demonstrated that the mutant p53-281G trans-activates the promoters of the human epidermal growth factor receptor (EGFR) and the human multidrug resistance gene (MDR-1) [48]. Subsequent research further revealed another mechanism by which mutant p53 proteins upregulate the expression of chemokines, showing that they can directly bind to and activate the CXCL1 or GRO1 promoters in SW480 colon cancer cells, thereby enhancing the oncogenic potential of mutant p53 [49]. In a more recent study, Butera G. et al. used mass spectrometry to analyze the secretome of pancreatic cancer cells (PDACs) expressing p53R175H and p53R273H. They found that mutant p53 significantly upregulated the protein expression levels of 15 secreted proteins, including growth factor IGFBP1, histones (HIST1H3A, HIST1H4A, HIST2H2AA3, etc.), and endothelial protein receptor (EPCR). These highly expressed secreted proteins promote biological functions such as PDAC hyperproliferation, chemoresistance, inhibition of apoptosis and autophagy, cell migration, and epithelial–mesenchymal transition. Chromatin immunoprecipitation (ChIP) experiments revealed that mutant p53 proteins bind to the promoters of these secreted protein genes, suggesting that mutant p53 directly regulates the transcription of these proteins [50].

The interactions between mutant p53 and other transcription factors (TFs) are structurally diverse. One of the most well-studied mechanisms by which mutant p53 influences gene expression is through the co-aggregation and sequestration of two tumor suppressor family members, p63 and p73 [51,52,53,54]. p63 and p73 can form homotetramers and heterotetramers with each other but do not form heterotetramers with wild-type p53 [55]. However, mutant p53 interacts with these family members through its altered DNA-binding core domain [53,56]. The interaction between mutant p53 and p63/p73 affects the ability of these proteins to regulate their target genes, with effects that can be positive or negative depending on the promoter context [53]. Through interaction with p63, mutant p53 can inhibit TAp63, leading to a transcriptional profile that promotes invasion and metastasis. This involves various signaling pathways and gene regulation, including Sharp1, Cyclin G2, miR-155, and others [53,56,57,58,59]. Notably, mutant p53 can utilize p63 as a molecular chaperone, anchoring to the promoters of its target genes and altering gene expression profiles to promote tumorigenesis. In a study on lung cancer, H1299 lung cancer cells expressing *p53* hotspot mutations (R175H, R248Q, R248W, R249S, R273H, and R282W) were used to prepare conditioned media. It was found that conditioned media from H1299 cells with mutant *p53* enhanced the migratory capacity of breast epithelial cancer cells (ZR-75-1) and H1299 cells, suggesting that mutant *p53* regulates the secretion of pro-migratory proteins. Genomic microarray analysis revealed that mutant *p53* upregulated 59 genes, including METTL7B, GPR17, TMCC3, NR2F2, and INO80C. Further ChIP experiments showed that mutant p53 and p63 were co-recruited to the promoters of these upregulated genes, transcriptionally activating the pro-migratory proteins and thereby promoting their secretion [60].

Inflammation is considered a hallmark of cancer. During inflammation, the main players recruited to the tumor microenvironment include inflammatory cells and several biochemical inflammatory mediators, such as cytokines, chemokines, interleukins, and enzymes, which profoundly influence tumor development and progression. In the study by Wellenstein, M.D. et al., it was found that miR-34a was reduced and the Wnt pathway activated in breast cancer cells lacking *p53*. The conditioned media from these cells had increased Wnt levels, promoting the expression of IL-1β by adjacent tumor-associated macrophages (TAMs), recruiting neutrophils, forming systemic inflammation, and promoting tumor metastasis [34].

Furthermore, in chronic hepatitis, it was found that the deletion of p53-negative regulator MDM2 led to sustained p53 activation, inducing hepatocyte apoptosis and an increase in the senescence-associated secretory phenotype, leading to inflammation and accelerated hepatocarcinogenesis [61]. Ghufran, S.M. et al. found elevated VEGF levels in conditioned media from p53-deficient HCC cells, which activated the phosphorylation of VEGF receptor-2, promoting endothelial cell proliferation, migration, and tumor vascular network formation [62]. These studies collectively supp ort that mutant p53 proteins regulate the expression of inflammatory chemokines through various mechanisms, maintaining the inflammatory state of the tumor microenvironment and thereby promoting tumor invasion and metastasis.

### 2.2. Mutant p53 Regulates the Function of the Secretome Within the Tumor Stroma

Cancer-associated fibroblasts (CAFs) are key components of the tumor microenvironment, primarily exerting their tumor-promoting effects through the secretion of proteins. Increasing evidence suggests that the loss or mutation of p53 affects the properties of surrounding cancer cells by modulating the proteins secreted by CAFs.

Hippokratis Kiaris et al. mixed *p53*-deficient fibroblasts with breast cancer cells (MCF7) and injected them into wild-type mice via the tail vein, observing an increase in the number of lung metastases in the mice [63]. Yoseph Addadi et al. found that, compared to *p53* wild-type mouse embryonic fibroblasts (MEFs), MEFs harboring the *p53^R172H^* mutation significantly promoted tumor growth [64]. Further studies revealed that mutant *p53* upregulated the expression of CXCL12 and CXCL1, two chemokines associated with tumorigenesis, in fibroblasts [64]. Similarly, proteins secreted by *p53*-deficient hepatic stromal cells (hepatic stellate cells), such as IL-4, induced the polarization of macrophages towards the tumor-promoting M2 type, thereby promoting liver cancer development [65]. Furthermore, co-culturing normal fibroblasts (NFs) with lung cancer cells resulted in the conformational change of p53 from wild-type to mutant form, regulating the secretion of 58 proteins, including matrix metalloproteinases (MMPs), which degrade the extracellular matrix and promote tumor progression [66].

Recent research indicates that mutant p53 can be transferred from tumor cells to fibroblasts via vesicles, activating their CAF characteristics and upregulating the secretion of proteins such as CCL2, CXCL1, G-CSF, GM-CSF, IL-1β, and IL-6 [67]. Another study confirmed the secretory nature of mutant p53, revealing that secreted mutant p53 can inhibit glycolysis, leading to the reduction in and dysfunction of CD4+ T lymphocytes, thus altering the tumor immune microenvironment [68]. Intervening in this secretory behavior of mutant p53 may represent a novel strategy for cancer treatment [69].

## 3. Mutant p53 Is Involved in Regulating the Pathways of Protein Secretion

The secretion of proteins can be categorized into ER–Golgi-dependent classical secretory pathways (SP) and ER–Golgi-independent unconventional protein secretion (UPS) pathways. Based on whether an N-terminal signal peptide is involved in the protein secretion process, secreted proteins are transported via different secretion pathways [70].

### 3.1. Mutant p53 Regulates the Tumor Secretome Through the SP

**Figure 2:**
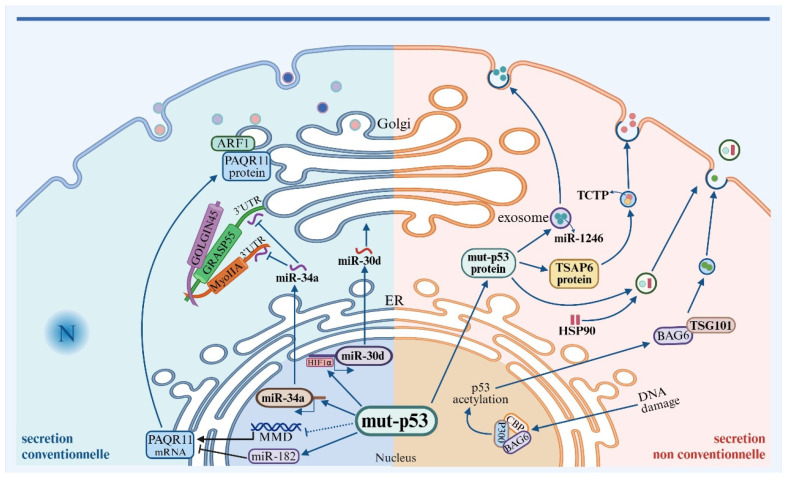
The mechanism of mutant p53 in regulating the secretome of tumors.

In the classical secretory pathway, the Golgi apparatus serves as the central hub for intracellular protein sorting and transport, primarily involved in post-translational modification, classification, and transportation of proteins. In vertebrates, the Golgi apparatus is composed of stacked flattened cisternae, known as Golgi stacks, which are interconnected to form a complete Golgi ribbon [71]. Vesicles are the main carriers of protein transport within cells, synthesized and loaded with proteins in the endoplasmic reticulum and Golgi apparatus, and subsequently transported to the plasma membrane and other intracellular membrane-bound organelles, maintaining membrane flow and facilitating protein transport [72,73]. This membrane dynamics enables cells to adapt to changes in the intracellular and extracellular environment, such as cell division, increased demand for secreted proteins, or maintaining plasma membrane integrity, ensuring cell survival [74,75]. Therefore, the structural organization of the Golgi ribbon is strictly regulated within cells. Mutant p53 plays a significant role in regulating the tumor secretome by altering the structure of the Golgi apparatus (Figure 2).

Mutant p53 regulates Golgi-associated proteins. In *p53*-deficient lung adenocarcinoma cells, there is a high expression of G55 (Golgi reassembly and stacking protein 55 kDa) [76]. G55 is a Golgi reassembly and stacking protein that forms a complex with Golgin45 (G45) and myosin IIA, maintaining Golgi integrity. It binds with various rab GTPases, facilitating the budding and formation of secretory vesicles within the Golgi, promoting secretion [77]. Clinically, high expression of G55 correlates positively with the malignancy of lung adenocarcinoma. Further research identified a binding site for miR-34a in the 3′-untranslated region (3′-UTR) of G55 mRNA. miR-34a binds to G55 mRNA, inhibiting its transcriptional activity. The loss of p53 downregulates miR-34a, relieving its inhibitory effect on G55 and myosin IIA, thereby promoting the secretion of two pro-tumorigenic proteins, SPP1 (Secreted Phosphoprotein 1) and IGFBP2 (Insulin-Like Growth Factor Binding Protein 2) [76].

Additionally, Capaci V et al. identified another mechanism by which mutant *p53* regulates Golgi-associated proteins in various malignant tumor cells with different p53 mutations (breast cancer MDA-MB-231/*p53^R280K^*, SK-BR-3/*p53^R175H^*, prostate cancer DU145/*p53^P223L^*^,V274F^, and colorectal cancer HT-29/*p53^R273H^*). Mutant p53 can bind to HIF1α, forming a transcriptionally active complex at the promoter region of miR-30d, thereby activating the transcription of miR-30d. miR-30d downregulates the expression of DGKZ (Diacylglycerol Kinase Zeta), leading to the accumulation of DAG (diacylglycerol) in the Golgi membrane, as it cannot be converted to phosphatidic acid. On one hand, accumulated DAG induces vesiculation of the Golgi, accelerating the transfer of secreted proteins from the cis-Golgi to the trans-Golgi [78]. On the other hand, DAG activates the Golgi-resident protein kinase D (PKD), promoting vesicle transport and increasing the release of ECM components such as fibronectin (FN), laminin V, and laminin B1, facilitating ECM deposition and remodeling, affecting mechanosignaling and stromal cell activation within the tumor microenvironment, thereby enhancing tumor growth, metastasis, and colonization [79,80].

Mutant p53 regulates the transcription of Golgi structural proteins. In A549 lung adenocarcinoma cells, CRISPR-Cas9-mediated knockout of wild-type *p53* resulted in the transcriptional activation of MMD, upregulating the expression of PAQR11 (Progestin and adipoQ receptor 11) [81]. PAQR11, encoded by the MMD gene, is a Golgi membrane protein localized on the trans-Golgi. PAQR11 recruits a protein complex containing ARF1 (Adenosine diphosphate ribosylation factors 1) to the trans-Golgi. ARF1, when bound to guanosine triphosphate (GTP), initiates Golgi membrane deformation, recruiting cargo adaptors and vesicular coat proteins to the Golgi membrane, generating bio-vesicles containing secreted proteins [82,83], promoting the secretion of protein kinase PLAU. Secreted PLAU activates the PLAU receptor/STAT3 (signal transducer and activator of transcription 3) axis, upregulating PQAR11 through feedback. Secreted PLAU inhibits anoikis in lung adenocarcinoma cells (344SQ), enhancing the stemness and anchorage-independent growth of tumor cells, promoting tumor metastasis [81].

### 3.2. Mutant p53 Regulates the Tumor Secretome Through UPS Pathways

Many secreted proteins lack N-signal peptides and therefore cannot bind to signal recognition particles (SRPs) to enter the endoplasmic reticulum. Consequently, they bypass the ER–Golgi system and are released through unconventional protein secretion (UPS) pathways. The unconventional protein secretion pathway includes vesicular and non-vesicular routes. The vesicular pathway is the primary route within UPS, relying on certain transmembrane protein families (e.g., TMED10, TMP21) to load secreted proteins into vesicular carriers, which are then delivered to the plasma membrane for release [84,85]. The mechanism of extracellular vesicle formation varies, and they are categorized based on their diameter into exosomes, microvesicles, and apoptotic bodies, playing a crucial role in intercellular communication [86,87,88,89]. Mutant p53 participates in vesicle biogenesis, altering the secretome of tumor cells and regulating signal transduction between tumor and stromal cells [90].

Exosomes are vesicles with diameters ranging from 30 to 150 nm, composed of a lipid bilayer containing membrane proteins, and displaying specific markers on their surface (e.g., Her2/Neu tumor antigen, Mart1 tumor antigen) [91]. Exosomes transfer RNA and proteins to neighboring or distant cells, regulating host cell physiological functions such as antigen presentation to activate immune responses and inhibiting tumor apoptosis [92,93]. p53 is involved in regulating the production and cargo of exosomes [94,95]. In mouse fibroblasts and bone marrow-derived dendritic cells (BMDCs), damage-activated p53 promotes exosome release, whereas the number of exosomes is significantly reduced in p53-deficient cells [96]. Alexandra Pritchard et al. found that conditioned media from wild-type p53 adenocarcinoma alveolar epithelial cells (A549) contained larger and more concentrated exosomes compared to p53-deficient lung cancer cells (H358), further confirming the role of p53 in regulating exosome release. Another study showed that exosomes mediated by mutant p53 could induce bone marrow (BM) cells and myeloid-derived suppressor cells (MDSCs) to differentiate into M2 macrophages, promoting immune evasion of lung cancer cells. However, the molecular mechanisms by which p53 and mutant p53 promote exosome release are not yet fully elucidated [97]. Preliminary mechanistic studies suggest that p53 upregulates the transcription of TSAP6 (tumor suppressor-activated pathway 6) protein, which co-localizes with translationally controlled tumor protein (TCTP) in cellular vesicles, promoting TCTP secretion and inducing anti-tumor immunity. TSAP6 is a six-transmembrane protein involved in vesicle transport and protein secretion, located in vesicular structures [98].

Yulin Sun et al., discovered in colorectal cancer research that HGS (hepatocyte growth factor-regulated tyrosine kinase substrate) is a key protein mediating exosome release by mutant p53. Colorectal cancer cells with p53 deletion or the p53R273H mutation exhibit low HGS expression, leading to smaller diameter exosomes and reduced secretion dependent on HGS [99]. Clinically, low HGS expression is positively correlated with the malignancy of colorectal cancer. A study from the NIH revealed that in colorectal cancer cells, mutant *p53* (HCT248^R116W^, H157^V175F^, H127^R273H^) promotes the secretion of miR-1246-positive exosomes. Mutant p53 enhances the SUMOylation of hnRNPa2b1 (Heterogeneous nuclear ribonucleoproteins A2/B1, a highly expressed RNA-binding protein involved in various processes of RNA metabolism including mRNA/miRNA synthesis and RNAm6A modification binding). SUMOylated hnRNPa2b1 recognizes and binds to specific short sequences (GGAG and CCCU) on miR-1246, loading miR-1246 into exosomes and promoting their extracellular secretion. miR-1246+ exosomes are internalized by adjacent macrophages, inducing the polarization of macrophages from M1 to M2, and secreting pro-tumor factors such as IL-10, TGF-β, and MMP, thus inducing an anti-inflammatory tumor microenvironment and promoting tumor progression [100].

Microvesicles, with diameters between 100 and 1000 nm, are involved in the transport of various molecules such as miRNA, mRNA, DNA, and functional proteins [101,102,103,104], and serve as an important means of extracellular communication [105]. Given the significant biological functions of microvesicles in tumor immune evasion and metastasis, the synthesis and release of vesicles have become key research focuses.

p53 directly participates in the formation of microvesicles. In melanoma migration mechanism studies, mutant p53 was found to be involved in the formation and release of microvesicles [106]. Mechanistic studies suggest that during DNA damage, BAG6 (HLB-A-associated transcription factor 3) enters the nucleus and forms an acetyltransferase complex with CBP/P300, recruiting p53 and promoting p53 acetylation, which enables BAG6 to enter the cytoplasm. Nuclear BAG6, relying on its P(S/T)AP motif, binds to TSG101 (an ESCRT protein complex that remodels the inner membrane, leading to membrane budding and vesicle formation), promoting the release of BAG6+ vesicles with anti-metastatic activity and inhibiting melanoma cell lung metastasis.

Moreover, mutant p53 can be loaded into microvesicles, promoting the activation of cancer-associated fibroblasts (CAFs). In six common malignancies carrying mutant p53 (breast cancer, prostate cancer, ovarian cancer, uterine cancer, lung cancer, and colorectal cancer), mutant p53 binds to HSP90 and enters microvesicles. Fibroblasts capturing mutant p53-carrying microvesicles activate CAF characteristics via Nrf2 [67].

Apoptotic bodies are vesicles with diameters ranging from 500 to 4000 nm, generated during programmed cell death. Currently, there are no reports on the relationship between mutant p53 and apoptotic bodies. Whether mutant p53 is involved in regulating the formation of apoptotic bodies remains to be further investigated.

## 4. Tumor Secretome Feedback Regulates Tumor Microenvironment

The tumor microenvironment (TME) refers to the complex and heterogeneous multicellular milieu that surrounds tumor cells during tumorigenesis. The TME typically includes immune cells such as T and B lymphocytes, tumor-associated macrophages (TAMs), dendritic cells (DCs), natural killer (NK) cells, neutrophils, and myeloid-derived suppressor cells (MDSCs), which collectively contribute to the regulation of tumor immune escape [107,108]. Stromal cells, including cancer-associated fibroblasts (CAFs), pericytes, and mesenchymal stromal cells, serve as key components of the TME, performing various functions such as matrix deposition and remodeling, extensive crosstalk with cancer cells, and interaction with infiltrating leukocytes [109]. The extracellular matrix (ECM), as a critical structural scaffold, provides support for tumor cell invasion and chemotaxis [110,111], while blood vessels supply essential nutrients and oxygen to the tumor [112], and lymphatic vessels contribute to immune regulation [113]. These components interact with each other and with cancer cells through intricate signaling networks, collectively maintaining tumor progression.

According to existing reports, mutations in p53 often affect the tumor secretome, with significant consequences for the tumor microenvironment (TME). In a study conducted by Julie M, tumor-associated macrophages (TAMs) were established in vitro, and Nutlin-3 was used to activate the p53 protein. This activation cooperated with NF-κB to target the IL-6 promoter region, promoting its transcription and expression. Concurrently, the mRNA levels of other pro-inflammatory factors, including TNF-α, IL-8, CXCL1, and CCL20, were significantly elevated, and the protein levels of related inflammatory cytokines were increased in the macrophage secretome [114]. IL-6 is a central molecule in tumor-related inflammation, and its downstream STAT3 signaling pathway is considered a key driver of cancer-associated inflammation. It is often activated in malignant tumor cells, facilitating extracellular matrix degradation, tumor angiogenesis, endothelial cell activation, and enhancing tumor cell migration [115]. Research by Cooks et al. also demonstrated that mutant *p53* (HCT248^R116W^, H157*^V175F^*, H127*^R273H^*) drives the secretion of miR-1246-enriched exosomes, which are internalized by adjacent macrophages. This internalization leads to the polarization of macrophages from M1 to M2, resulting in the secretion of pro-tumor factors such as IL-10, TGF-β, and MMPs, which promote an anti-inflammatory TME and foster tumor progression [100]. In a more recent study, Nian et al. found that in p53-deficient hepatocellular carcinoma (HCC), cancer stem cells secrete large amounts of IL-34 into the surrounding environment, which induces the expression of CD36 in macrophages and stimulates fatty acid oxidation. This process promotes the polarization of macrophages to M2-type macrophages, forming tumor-associated macrophages that suppress T cell-mediated anti-tumor immunity and facilitate immune evasion, thereby promoting tumor progression [116]. These findings collectively support the role of mutant p53 in modulating the tumor secretome, influencing immune cells within the TME, maintaining an inflammatory microenvironment, and thereby promoting tumor invasion and metastasis.

In addition to the immune microenvironment, the presence of cancer-associated fibroblasts (CAFs) and extracellular matrix (ECM) also plays a critical role in tumor progression. A study by Ma et al. confirmed that exosomes containing mutant p53 secreted by tumor cells can activate PDGF and TGF-β signaling pathways in normal fibroblasts. This activation leads to the transcription of Nrf2 target genes, driving the transformation of normal fibroblasts into CAFs [67]. Additionally, research by David Novo and colleagues highlighted that exosomes derived from tumor cells expressing mutant p53 can regulate the endocytosis and recycling of α5β1 integrins in fibroblasts through a Rab-coupling protein (RCP)/diacylglycerol kinase α (DGKα)-dependent pathway. This modulation affects the organization and adhesive properties of ECM deposited by fibroblasts [117].

Tumors are complex conglomerates, and the aforementioned studies underscore the diverse ways in which mutant p53 influences the tumor secretome. Ultimately, these effects are communicated through signaling pathways that impact the entire tumor. Targeted therapeutic strategies could therefore be developed to either directly target mutant p53 or selectively inhibit the mechanisms by which mutant p53 regulates the tumor secretome.

## 5. Targeted Therapy for Mutant p53

Given the malignant roles of mutant p53 in various tumors and diseases, targeting mutant p53 is of paramount importance for cancer treatment. Currently, strategies to target mutant p53 are categorized into the following approaches: (1) Direct targeting of mutant p53: utilizing small molecules to restore or stabilize p53 function. (2) Indirect targeting: focusing on signaling pathways altered by p53 mutations, such as YAP/TAZ or other related genes. (3) Synthetic lethality: exploiting gene deficiencies caused by p53 mutations to induce cancer cell death by targeting other genes. (4) Non-coding RNAs: studies have shown that interfering with non-coding RNAs can exert anti-tumor effects on p53-mutant cancer cells. (5) Restoring the wild-type conformation of mutant p53.

Research by PMV Pharmaceuticals has found that the small molecule PC14586 specifically binds to the *p53^Y220C^* mutant pocket, restoring its tumor-suppressive function. This is the first *p53^Y220C^* reactivator to enter clinical trials [118]. PANDA (P21-associated ncRNA DNA damage-activated) is a long non-coding RNA (lncRNA) activated after DNA damage. It is associated with the stabilization of the p53 tumor suppressor protein. Studies have shown that PANDA stabilizes p53 by inhibiting the function of nuclear transcription factor Y (NF-YA) and suppressing apoptosis following DNA damage [119]. Kong et al. developed a novel PROTAC (Proteolysis-Targeting Chimera) that precisely targets the p53-R175H mutant for degradation, providing a new strategy for treating tumors harboring this specific mutation [120]. The small molecule probe U7D-1 degrades USP7, indirectly affecting the interaction between MDM2 and p53, which may exert anti-tumor effects on p53-mutant cancers [121]. Additionally, compounds such as arsenic trioxide (ATO) [122], phenethyl isothiocyanate (PEITC) [123], and COTI-2 [124,125] are capable of targeting mutant p53 by restoring its wild-type conformation.

Although several targeted therapies for mutant p53 have been developed, and some have entered clinical stages, no FDA-approved drugs specifically targeting p53 mutations currently exist. However, certain drugs have shown potential in clinical trials, such as Lonsurf (TAS-102) and Talzenna (Talazoparib) [126]. These drugs have been used individually to treat cancer and demonstrated effectiveness against TP53-mutant cancer cells in animal models.

Furthermore, many drugs or therapeutic targets begin with in vitro cell validation or in vivo mouse model validation, which still presents significant heterogeneity compared to human bodies. Accelerating the clinical translation of existing drugs is a critical task. Various preclinical models developed so far have greatly facilitated clinical translation [127]. However, the limitations of single models cannot simulate the complete and authentic tumor environment. In recent years, laboratories have employed various methods, including co-culture systems to mimic the supportive role of the tumor microenvironment, spatial transcriptomics to reveal the cell symbiosis state within the tumor microenvironment, and 3D bioprinting to simulate tumor structures. These approaches aim to integrate different systems, expand their application fields based on independent models, and reproduce the true complexity of tumors, including tumorigenesis and progression processes [128]. These advancements could significantly reduce resource and time costs in clinical trials, expedite the clinical translation of drugs, and provide new possibilities for cancer treatment strategies.

## 6. Conclusions

Increasing evidence suggests that tumor cells do not act in isolation during their progression to malignancy. The tumor microenvironment plays a crucial role in promoting tumor growth, invasion, and metastasis, and its formation is induced by the tumor itself. As the most frequently mutated tumor suppressor gene, *p53*, is highlighted in this study, we observe how mutant p53 regulates the tumor secretome through various mechanisms, affecting the synthesis and release of secreted proteins, mediating signal transduction between tumors and stromal cells, inducing the tumor microenvironment, stimulating inflammatory cancer-associated microenvironments, and promoting tumor progression [129].

Different types of mutant p53 have varying impacts on cell cycle regulation, apoptosis, and DNA repair (Table 1), and different types of p53 mutations can influence the tumor cell secretome and its microenvironment in various ways. These mutations may lead to changes in the expression of growth factors, cytokines, and other molecules within the secretome, consequently affecting extracellular matrix remodeling, angiogenesis, and the recruitment and function of immune cells in the tumor microenvironment. For example, certain p53 mutations can enhance the secretion of pro-inflammatory factors, increasing tumor invasiveness and metastatic potential [114,116,130]; simultaneously, mutant p53 alters interactions between tumor cells and surrounding stromal cells, impacting immune responses and promoting immune evasion [116,131,132]. Moreover, ECM proteins are continuously assembled and remodeled within tumors, with integrins regulating ECM properties through endosomal transport [133,134]. The transfer of mutp53-containing exosomes can reprogram integrin transport in fibroblasts, leading to ECM remodeling and pre-metastatic niche formation, thereby promoting metastatic invasion of tumor cells [117]. Therefore, understanding the heterogeneity of p53 mutations and their effects on the secretome and microenvironment is crucial for developing personalized therapeutic strategies.

Targeting mutant p53 or its regulated signaling pathways to obtain therapeutic benefits mainly includes restoring the function of mutant p53, inhibiting the harmful effects of mutant p53, and using mutant p53 as a biomarker to guide treatment. Restoring the function of mutant p53 can be achieved by designing small molecules that refold it into its wild-type conformation or enhancing the transcriptional activity of mutant p53. Inhibiting the harmful effects of mutant p53 involves developing drugs to block its interactions with other proteins or promoting its degradation. Additionally, as the presence of mutant p53 is often associated with specific biomarkers, they serve as potential predictive indicators for therapeutic response, enabling precision medicine.

## Figures and Tables

**Table 1:** Functional heterogeneity and unique roles of different p53 mutation types.

Type	Mutation Site	Main Function	Unique Effect
Contact mutation	*p53^R248Q^* [135]; *p53^R273H^* [136]	Loss of DNA binding, partial retention of protein interaction ability	Cell invasion, angiogenesis, anti-apoptosis, genomic instability
Conformational changes	*p53^R175H^* [137]; *p53^G245S^* [138]	Destroy protein structure, lose tumor suppressor function, GOF activity	Immune escape, metabolic reprogramming, epigenetic regulation and stem cell characteristics
	*p53^Y220C^* [118]	Protein cleft leads to inactivation, but can be partially repaired	Small molecule targets to regulate inflammatory signals
Others	*p53^R282W^* [139]; *p53^R249S^* [140]	Loss of DNA activity and altered downstream effects	Specific carcinogenic effects related to metabolic regulation and environmental exposure
